# Evaluation of a comprehensive cardiovascular magnetic resonance protocol in young adults late after the arterial switch operation for *d*-transposition of the great arteries

**DOI:** 10.1186/s12968-014-0098-5

**Published:** 2014-12-11

**Authors:** Daniel Tobler, Manish Motwani, Rachel M Wald, Susan L Roche, Flavia Verocai, Robert M Iwanochko, John P Greenwood, Erwin N Oechslin, Andrew M Crean

**Affiliations:** Toronto Congenital Centre for Adults, Peter Munk Cardiac Centre, University Health Network, Toronto General Hospital, 585 University Avenue, 5 N-525, Toronto, ON M5G 2N2 Canada; Department of Cardiology, University Hospital Basel, Basel, Switzerland; Multidisciplinary Cardiovascular Research Centre, Leeds Institute of Genetics, Health and Therapeutics, University of Leeds, Leeds, UK; Department of Medical Imaging, Toronto General Hospital, Toronto, Canada

**Keywords:** Transposition of the great vessels, Cardiovascular magnetic resonance, Nuclear cardiology, Ischemia

## Abstract

**Background:**

In adults with prior arterial switch operation (ASO) for *d*-transposition of the great arteries, the need for routine coronary artery assessment and evaluation for silent myocardial ischemia is not well defined. In this observational study we aimed to determine the value of a comprehensive cardiovascular magnetic resonance (CMR) protocol for the detection of coronary problems in adults with prior ASO for *d*-transposition of the great arteries.

**Methods:**

Adult ASO patients (≥18 years of age) were recruited consecutively. Patients underwent a comprehensive stress perfusion CMR protocol that included measurement of biventricular systolic function, myocardial scar burden, coronary ostial assessment and myocardial perfusion during vasodilator stress by perfusion CMR. Single photon emission computed tomography (SPECT) was performed on the same day as a confirmatory second imaging modality. Stress studies were visually assessed for perfusion defects (qualitative analysis). Additionally, myocardial blood flow was quantitatively analysed from mid-ventricular perfusion CMR images. In unclear cases, CT coronary angiography or conventional angiography was done.

**Results:**

Twenty-seven adult ASO patients (mean age 23 years, 85% male, 67% with a usual coronary pattern; none with a prior coronary artery complication) were included in the study. CMR stress perfusion was normal in all 27 patients with no evidence of inducible perfusion defects. In 24 cases the coronary ostia could conclusively be demonstrated to be normal. There was disagreement between CMR and SPECT for visually-assessed perfusion defects in 54% of patients with most disagreement due to false positive SPECT.

**Conclusions:**

Adult ASO survivors in this study had no CMR evidence of myocardial ischemia, scar or coronary ostial abnormality. Compared to SPECT, CMR provides additional valuable information about the coronary artery anatomy. The data shows that the asymptomatic and clinically stable adult ASO patient has a low pre-test probability for inducible ischemia. In this situation it is likely that routine evaluation with stress CMR is unnecessary.

## Background

Good long-term outcome in adults after the arterial switch operation (ASO) has been reported [1]. In historical cohorts, there were intermittent cases after ASO of ostial coronary stenosis and ischemic symptoms occurring as a result of coronary distortion at the re-implantation site [2]. In those early cohorts, cardiovascular events have been reported in up to 7% of childhood ASO survivors, most of these occurred early after the operation [3]. In the current era, late coronary complications are rare in both, children and adults [4]. Nevertheless, evaluation of inducible ischemia in asymptomatic adult ASO patients is controversial. Non-invasive testing for myocardial ischemia, usually performed by stress echocardiography or nuclear imaging techniques, has shown insufficient sensitivity [3].

Cardiovascular Magnetic Resonance (CMR) has emerged as the reference standard for non-invasive imaging of patients with many forms of congenital heart disease [5]. Because children and adults with congenital heart disease often need serial imaging, CMR is the ideal modality to screen and to follow these complex patients due to its lack of ionizing radiation [6]. Furthermore, the recently published CE-MARC trial has established the high diagnostic accuracy of stress CMR in coronary heart disease [7]. Therefore, the objective of this study was to determine the value of a comprehensive CMR protocol in the detection of coronary problems in an adult ASO population.

## Methods

### Patients

After institutional research ethics board approval, adult ASO survivors (≥18 years) were recruited consecutively during routine clinic visits and studied between March 2009 and July 2013. Patients with a permanent pacemaker were excluded from the study. All patients gave informed written consent.

**Figure 1:**
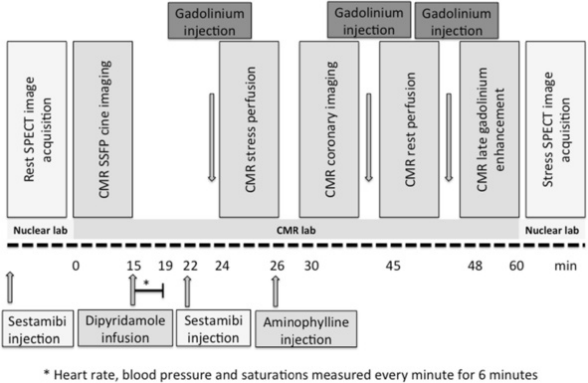
**Schematic demonstrating the timeline of the integrated CMR/SPECT assessment of myocardial function, perfusion, scar and coronary anatomy.**

Patients underwent a comprehensive CMR protocol including rest and stress perfusion, Late Gadolinium Enhancement (LGE), CINE functional analysis and coronary CMR for the detection of occult coronary lesions. Additionally, SPECT imaging was done on the same day as a confirmatory second imaging modality. Figure 1 details the combined single stress - dual perfusion SPECT/CMR protocol.

### Cardiovascular magnetic resonance

All patients underwent CMR on a 1.5 T scanner (Siemens Avanto, Siemens Medical Systems, Erlangen, Germany). After standard localizer sequences, steady state free precession (SSFP) images were acquired in the axial and short axis planes providing full ventricular coverage (6 mm slice thickness, 2 mm interslice gap, TR 35-50 ms, reconstructed to 25 frames per cardiac cycle). LGE images were acquired 10 minutes after the injection of a total dose of 0.2 mmol/kg of gadodiamide using a standard segmented inversion recovery gradient echo sequence at slice locations matched to the short axis SSFP images. Whole heart CMR angiography was performed as an axial block acquired in 1 mm thick partitions using a free-breathing navigated 3D SSFP sequence (end-diastolic trigger, in-plane resolution of 1.0-1.5 × 1.0-1.5 mm, gate and drift on, central acquisition window ±2.5 mm). Additional high resolution SSFP cine imaging targeted to the coronary ostia was used in selected cases (1.0 × 1.0 × 2.5-3.0 mm voxel size, TR 50-70 ms).

### CMR post processing

Short axis SSFP stacks were manually contoured at end-systole and end-diastole using offline post processing software (cmr42, Circle Cardiovascular Imaging, Calgary, Canada) and volumes and ejection fraction calculated using Simpson’s rule of discs. Endo- and epicardial contours of the short axis turboflash LGE image stack were traced and percentage of enhanced myocardium was defined as that exceeding a signal intensity of 5SD from a region of interest placed in remote normal myocardium. Whole heart coronary CMR volumes were examined at native resolution and plane of acquisition as well as following centre-line reformat and creation of orthogonal maximum intensity projection views (Vitrea workstation, Vital Images, Minneapolis, USA).

### Myocardial blood flow estimation by CMR

Perfusion images were processed offline using previously validated in-house software (PMI 0.4; written in IDL 6.4 (ITT Visual Information Systems, Boulder, CO) [8]. Following manual rigid motion-correction, a circular region of interest was drawn in the basal left ventricular cavity to derive the arterial input function. A myocardial circular region of interest excluding any dark-rim artifact was drawn for the entire myocardium in the mid-ventricular slice. Signal intensity–time data were converted to concentration-time data by subtracting the baseline signal, and myocardial blood flow was estimated at stress and rest using constrained deconvolution with a delayed Fermi-model applied to the first pass [9-11]. Myocardial perfusion reserve was calculated as stress myocardial blood flow divided by rest myocardial blood flow.

### Technetium^99m^ Sestamibi™ image acquisition and analysis

Images were acquired on a Philips Forte (Philips Medical Systems, Netherlands) fitted with a high resolution VXHR collimator. Prior to SPECT image acquisition, a 30 second left anterior oblique image was acquired with the counts derived from the most count-dense pixel used to determine the time per frame for the SPECT image. Data were acquired in roving field of view step and shoot mode with 3 degree angular steps. Attenuation-corrected data were reconstructed iteratively and subsequently re-binned to create 8 cardiac phases for function and volume assessments. Summed stress, rest and difference scores (SSS, SRS, SDS) were calculated from segmental defect size using a commercially available package (QPS, Cedars-Sinai, Los Angeles, USA).

### Combined single stress – dual perfusion protocol

Patients were instructed to withhold all caffeinated products for 24 hours prior to stress imaging. Prior to CMR, patients underwent rest perfusion imaging in the nuclear laboratory following injection of 300Mbq of technetium^99m^ methoxyisobutylisonitrile (Sestamibi™). Four hours later the patient was transferred to the CMR suite where vasodilator stress was performed in the magnet with a 4 minute infusion of dipyridamole at a total dose of 0.56 mg/kg. At 6 minutes from the start of infusion (maximal hyperemia) the patient was given a bolus of 650Mbq intravenous Sestamibi™ followed by a saline flush. The i.v line was then reconnected to the power injection and a bolus of 0.05 mmol/kg gadodiamide injected at 5 ml/sec followed by a saline flush of 25 ml at 5 ml/sec. An saturation recovery gradient echo sequence was used to acquire 3 short axis slices (base, mid ventricle and apex) every heart beat (8 mm slice thickness, in plane resolution 1.5-1.8 × 1.5 × 1.8 mm, 80 cardiac phases). Once the stress portion was complete, dipyridamole was reversed in every case with a slow intravenous bolus of aminophylline 2 mg/kg up to a maximum dose of 200 mg, until complete relief of symptoms or return to baseline heart rate. After a minimum period of 20 minutes the rest perfusion CMR study was acquired with the same anatomic and technical parameters. At the end of the examination the patient was transferred to the nuclear laboratory where SPECT perfusion images pertaining to the in-magnet episode of stress were obtained. Time between in-magnet injection of Sestamibi™ during stress and SPECT image acquisition in the nuclear laboratory was set at 60 minutes.

### Statistical methods

Statistical analysis was performed using SPSS software version 20 (IBM SPSS 20, 2011) Descriptive statistics of continuous data are presented as mean ± standard deviation. Changes from baseline to hyperemic flow were compared by the paired Student's *t* test.

## Results

### Patient population

Of 65 adult ASO patients under active care in our center at the time of inclusion, 33 patients were approached consecutively and 28 agreed to participate. One patient was claustrophobic at the time of the CMR examination and no useful data were obtained. The CMR results are therefore based on 27 patients in total. Basic demographic data are provided in Table 1. Historically, at the Hospital for Sick Children in Toronto, ASOs were performed only in complex patients (with ventricular septal defect) until 1986. After 1986, the strategy changed and also simple TGA (with intact ventricular septum) were operated with the Jatene procedure. In our cohort, 2 patients with late repair had complex TGA and one patient had Taussig Bing anatomy (DORV with subpulmonary ventricular septal defect and TGA). In all of these 3 patients, a pulmonary artery banding was performed as palliation prior to ASO. Of study patients, 23 (85%) were male (compared to 62% in the entire cohort under active care, p = 0.03). Otherwise, patients studied by CMR did not differ in basic demographic details compared to ASO patients under active care (Table 2) [1].

**Table 1:** **Patient characteristics**

	**Early repair** * **n = 24**	**Late repair n = 3**
**Age at enrolment (years)**	21 ± 3	24 ± 8
**Male, n (%)**	21 (88)	2 (66)
**Age at surgery (days)**	6 ± 5	397 ± 592
**Era of ASO surgery**	1986 - 1991	1979 - 1985
**Cardiac anatomy**		
TGA with IVS	14	-
TGA with VSD	10	2
Taussig-Bing	-	1
**Additional lesions**		
Atrial septal defect	2	1
Coarctation	2	1
Subvalvular aortic stenosis	-	1
Subvalvular pulmonary stenosis	-	1
**Coronary pattern**		
1LCx2R (usual)	17	1
2RLCx (single ostium)	2	2
1L2RCx (CX from RCA)	4	
1Cx2RL (LAD from RCA)	1	
**Bypass time, min**	141 ± 99	203 ± 25
**Cross clamp time, min**	99 ± 22	55 ± 39
**Additional repair**		
Takeuchi repair	1	-
RV-PA conduit	-	1
Subclavian flap repair of coarctation	1	1
End-to-end repair of coarctation	1	-
**Indication for exam**		
Chest pain	5	2
Coronary surveillance	19	1
**Prior ischemia testing as a child** ^**†**^		
Yes	13	1
No	9	2
Unknown	2	-
**Type of prior ischemia testing**		
Treadmill	1	
Thallium	8	
Technetium	7	
Coronary angiography	3	

IVS = Interventricular septum; RV-PA = Right ventricular to pulmonary artery; TGA = Transpostion of the great arteries; VSD = Ventricular septal defect.

*Early repair defined as occurring within the first 30 days of life.

^**†**^Median number = 1 (range 1–3).

**Table 2:** **Comparison of patient characteristics between the studied cohort and the entire cohort of ASO patients under active care**

	**STUDY COHORT**	**TORONTO COHORT**	**p-value**
	**n = 27**	**n = 65**	
**Age at enrolment (years)**	21 ± 3*	24 ± 8	NS
**Male, n (%)**	23 (85)	40 (62)	0.03
**Age at surgery (days)**	6 ± 5*	6 ± 5	NS
**Era of ASO surgery**	1979 - 1991	1979 - 1991	
**Cardiac anatomy**			
TGA with IVS	14	36	NS
TGA with VSD	12	27	
Taussig-Bing	1	2	
**Coronary pattern**			
1LCx2R (usual)	18	42	NS
2RLCx (single ostium)	4	5	
1L2RCx (CX from RCA)	4	13	
1Cx2RL (LAD from RCA)	1	3	
**Bypass time, min**	141 ± 99*	150 ± 54	NS
**Cross clamp time, min**	99 ± 22*	93 ± 21	NS

ASO = arterial switch operation, Cx = circumflex artery; IVS = intact ventricular septum; LAD = left coronary artery; RCA = right coronary artery; TGA = transposition of the great arteries; VSD = ventricular septum defect.

*Patients with early repair only.

### CMR studies

The imaging protocol was completed successfully in all participants. Mean left ventricular and right ventricular ejection fraction were 55 ± 5% and 51 ± 7%, respectively and mean left and right end-diastolic volume assessment indexed to body surface area were 105 ± 22 ml/m^2^ and 113 ± 23 ml/m^2^, respectively.

**Figure 2:**
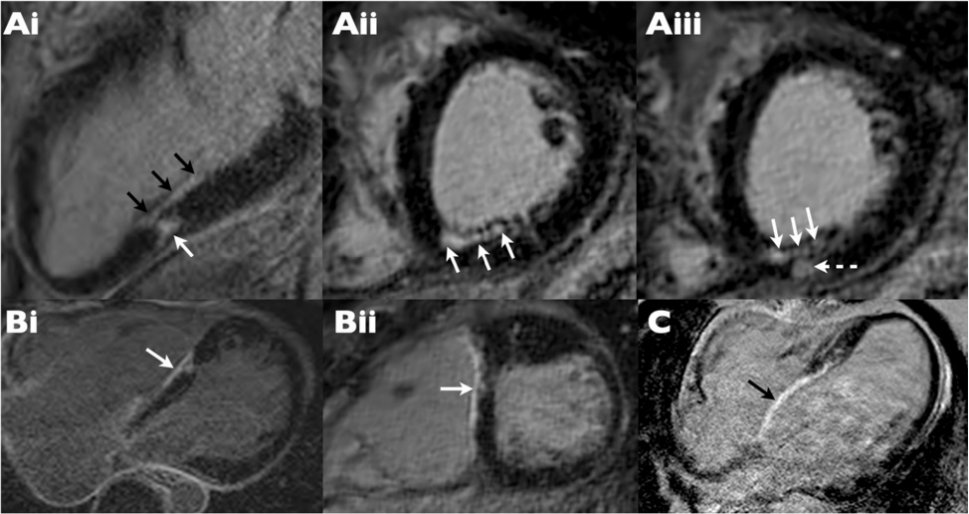
**Real and ‘pseudo-real’ late gadolinium enhancement (LGE) in 3 patients with positive LGE studies. (Ai-iii)** Genuine LGE is evident in the mid inferior wall in the 2-chamber view **(Ai)** with both linear (black arrows) and more focal (white arrow) enhancement. Confirmation of these findings is provided by short axis cross cuts through this region, which also show subendocardial (white arrows) and midwall nodular (dotted arrow) myocardial scar. **(Bi-ii)** “Pseudo” LGE (white arrow) is present at the site of ventricular septal defect (VSD) repair shown in 4-chamber **(i)** and short axis **(ii)** views. **(C)** “Pseudo” LGE (black arrow) evident in a large surgical patch placed for VSD repair.

Stress perfusion was visually normal in all participants with no evidence of inducible perfusion defects (Table 3) despite appropriate physiologic and symptomatic response to dipyridamole (mean rise in rate-pressure product from 8296 bpm*mmHg to 12389 bpm*mmHg, p < 0.01). Whole heart coronary MR angiography was successfully completed in 18 patients (67%) – in the remaining 9 cases, coronary ostial evaluation was instead performed with high resolution SSFP cine imaging due to either poor navigator efficiency with the whole heart sequence and/or constraints on available scan time. LGE images were acquired in all 27 patients but were abnormal in only 3 (Table 3) where very small volumes of enhancement were apparent (Figure 2). The ostia were seen and evaluated as normal in 24 cases, abnormal in 2 cases and non-diagnostic in one case (Table 3).

**Table 3:** CMR (n = 27) and SPECT (n = 25) findings*

	**Stress CMR**	**Coronary ostia**	**LGE**	**SPECT**
**Patient 1**	N	N	No	**A – SDS 3**
**Patient 2**	N	N	No	N
**Patient 3**	N	N	No	N
**Patient 4**	N	N	No	N
**Patient 5**	N	N	No	N
**Patient 6**	N	N	No	**A – SDS 2**
**Patient 7**	N	N	**Yes**	**A – SDS 2**
**Patient 8**	N	N	No	**E – SDS 0**
**Patient 9**	N	N	No	N
**Patient 10**	N	N	No	N
**Patient 11**	N	**A**	**Yes**	N
**Patient 12**	N	**A**	No	N
**Patient 13**	N	N	No	**E – SDS 0**
**Patient 14**	N	N	No	Declined
**Patient 15**	N	N	No	**A – SDS 3**
**Patient 16**	N	N	No	N
**Patient 17**	N	N	No	**A – SDS 2**
**Patient 18**	N	N	No	**A – SDS 2**
**Patient 19**	N	N	No	N
**Patient 22** ^**§**^	N	**A**	No	**A – SDS 4**
**Patient 23**	N	N	No	N
**Patient 25**	N	N	**Yes**	**A – SDS 0**
**Patient 26**	N	N	No	**E – SDS 0**
**Patient 27**	N	N	No	**E – SDS 0**
**Patient 28**	N	N	No	**A – SDS 5**
**Patient 29**	N	N	No	Declined
**Patient 30**	N	N	No	N

*Note that patients are not all numbered consecutively.

^§^Patient 22 was scored as having an abnormal proximal right coronary artery on CMR, however SPECT perfusion defect was in the LAD territory with normal left main ostium and no LGE on CMR (see text).

N = normal; A = abnormal; E = equivocal; LGE = late gadolinium enhancement; SDS = summed difference score; N/A = not available.

### Cases in which a coronary abnormality was recorded on CMR

**Figure 3:**
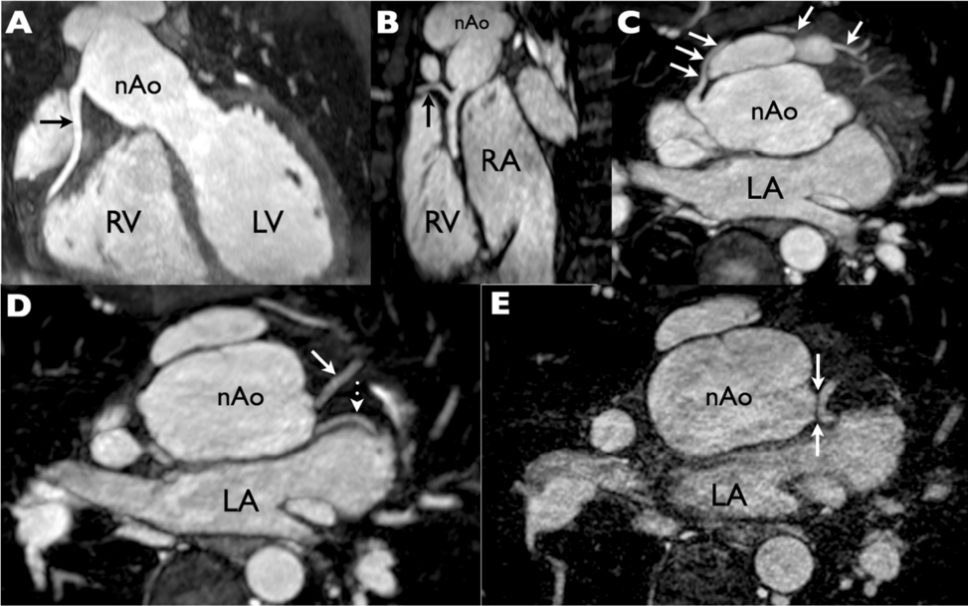
**Whole heart coronary magnetic resonance angiography (MRA) at 1×1×1 mm resolution with multiplanar maximum intensity projection reformats to demonstrate the coronary arteries. (A)** The right coronary artery (RCA) is seen to have a normal origin and course. **(B)** Excellent image quality is evident from the depiction of a small RCA marginal branch (arrow). **(C)** A large conal branch (arrows) takes a pre-pulmonic course and anastomoses with the left coronary system at the apex. **(D, E)** The left anterior descending (**D** solid arrow) and left circumflex (**D** dotted arrow) coronary arteries are shown in their proximal portions. Although the bifurcation of these vessels is clearly depicted, note that the left main coronary artery is not visible and that there is a’gap’ between the aortic root and the LAD/Cx bifurcation even on ultrahigh resolution (0.5 × 0.5 × 0.5 mm) MRA (E arrows). This was misinterpreted as an occlusion of the left main segment as the diagnosis of single coronary artery had not been recognized. RV = right ventricle; LV = left ventricle; nAo = neo-aorta; RA = right atrium; LA = left atrium.

In the first of the abnormal cases (Patient 12) the left main coronary artery could not be identified (Figure 3) – however it later transpired that the patient had previously been documented to have a single coronary system arising from the right coronary ostium.

**Figure 4:**
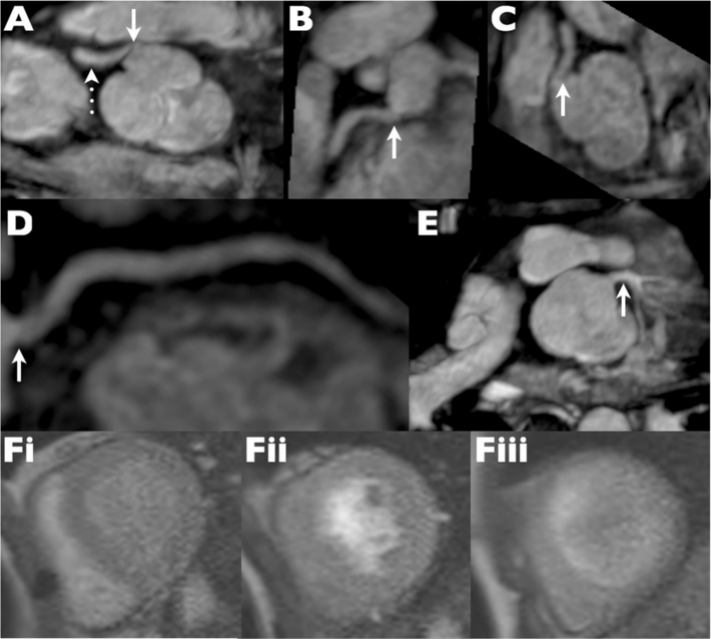
**Evaluation of a “kinked” right coronary artery. (A)** Straight axial image from a whole heart magnetic resonance angiogram demonstrates an apparent “kink” at the right coronary artery origin. **(B-D)** Subsequent multiplanar and centre line reformats however demonstrate that the ostium is in reality unobstructed. **(E)** The left coronary origin is also unobstructed. **(Fi-iii)** Stress perfusion magnetic resonance frames at basal, mid and apical left ventricular level show no evidence of any inducible perfusion defect. Quantitative perfusion measured in the right coronary territory was normal (not shown).

The second case was a patient with possible compression of the right coronary artery on CMR but negative CMR perfusion study (Patient 22). A small SPECT defect was recorded in the LAD territory and was therefore not congruent with the potential right coronary artery anomaly. Subsequent multiplanar reformats of the whole heart data set demonstrated tortuosity rather than obstruction (Figure 4). The patient remains asymptomatic and has declined further investigation by either CT coronary angiography or conventional catheterization.

**Figure 5:**
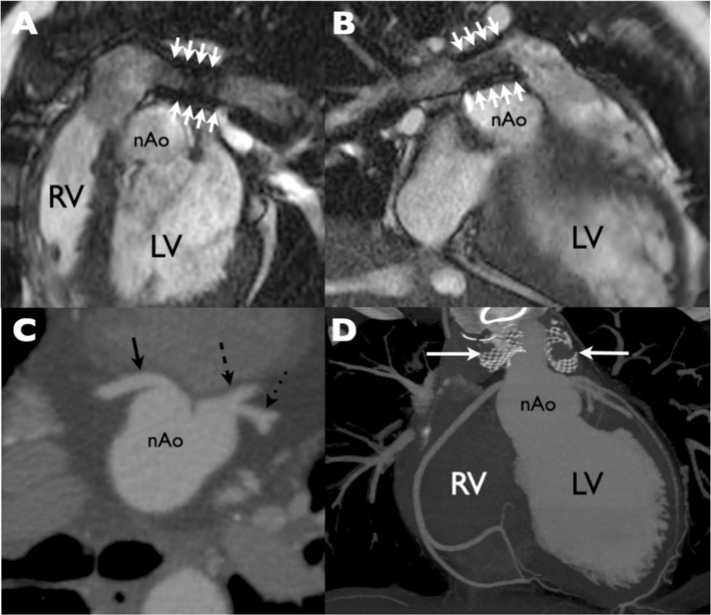
**Bilateral pulmonary artery stents obscuring coronary origins at CMR. (A, B)** Metallic artifact from bilateral pulmonary artery (PA) stents (arrows) on steady state free precession cine imaging obscures the coronary arteries as they emerge from the neo-aortic root. **(C)** Multiplanar reformat from low dose cardiac gated computed tomography reveals normal origins of the right coronary artery (solid arrow), left anterior descending (dashed arrow) and circumflex (dotted arrow) coronary arteries. **(D)** Lack of effect of bilateral PA stents (arrows) on visibility of the coronary arteries at cardiac CT is apparent on this coronal reformat. RV = right ventricle; LV = left ventricle; nAo = neo aorta.

In the third case (Patient 11), the ostia were unevaluable due to the presence of bilateral pulmonary artery stents (Figure 5); visual and quantitative CMR perfusion and SPECT were all normal and the subsequent coronary CT was unremarkable.

**Figure 6:**
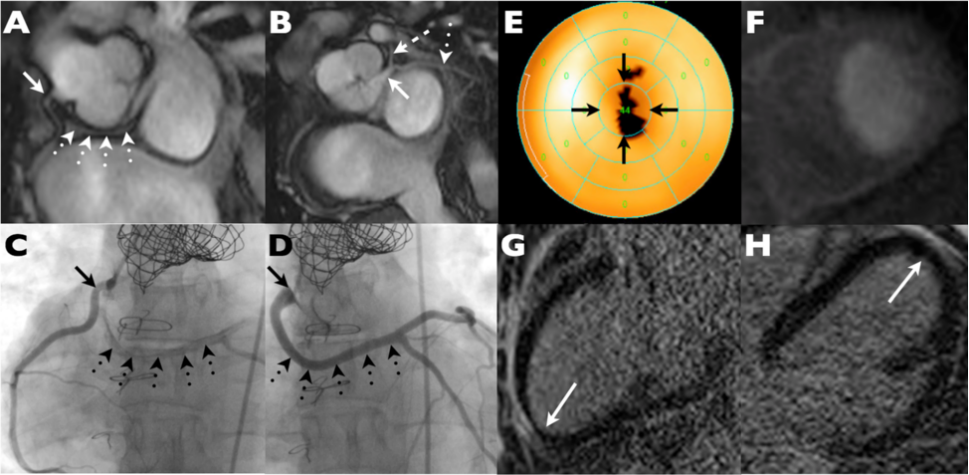
**Example of false positive SPECT study with a fixed apical defect. (A)** Steady state free precession imaging demonstrates a normal right coronary origin (solid arrow) but reveals a retro-aortic course of the left main coronary artery (dotted arrows). **(B)** The anomalous left main regains the normal position and bifurcates in to LAD (dashed arrow) and Cx (dotted arrow). **(C-D)** Conventional coronary angiography confirms undistorted coronary origins (solid arrows) and substantiates the presence of an anomalous left main coronary artery (dotted arrows). **(E)** Bullseye plot from resting SPECT indicates a fixed apical defect suggestive of infarction. **(F)** Stress perfusion CMR is normal. **(G, H)** Late gadolinium enhancement images in 2 and 4 chamber orientations show no evidence of any apical infarction.

In one additional case (Patient 4), stress CMR perfusion and whole heart coronary angiography were read as normal but - because of ongoing complaints of chest pain and a SPECT study read as mildly abnormal (SDS 2) - went on to conventional angiography, which confirmed undistorted coronary ostia and normal distal vessels (Figure 6).

### Quantitative stress perfusion CMR

There was a significant increase in absolute myocardial blood flow from the resting to vasodilated state (1.39 ± 032 ml/min/g at rest increasing to 3.21 ± 0.62 ml/min/g with stress, p < 0.0001). Nine patients had resting myocardial blood flow greater than 1.5 ml/min/g. Mean myocardial perfusion reserve for the cohort was 2.44 ± 0.77.

### Technetium 99 m Sestamibi™ perfusion studies

Twenty-five patients underwent rest-stress SPECT of whom 22/25 underwent the single stress dual perfusion (SSDP) protocol (Table 3). The remaining 3 patients returned for resting SPECT on another day. Two patients declined SPECT examination - one of whom subsequently underwent (normal) CT coronary angiography (Patient 14). Twelve patients were reported as having a normal SPECT scan (entirely normal in 10 cases (40%); probably normal in 2 cases (8%)). Four patients (16%) were reported as equivocal, and 9 (36%) were reported as abnormal. For these latter 13 patients the reported defects were relatively small with a mean summed stress score of 2.65 ± 3.55, mean summed rest score of 1.81 ± 3.36 and a mean summed difference score of 1.1 ± 1.61. One patient with an abnormal SPECT scan (SDS 0) subsequently had normal computed tomographic coronary angiography (Patient 25). Median effective dose was 11 mSv (range 9-14 mSv).

### Discordant CMR-SPECT studies

There was complete agreement between CMR and SPECT for the absence of visually assessed perfusion defects in 11 out of 24 (46%) paired comparisons and disagreement in 13 studies (54%). Disagreements were generally minor – in 5 out of 13 mismatches the SPECT SDS was zero, indicating that the defect seen at SPECT was not due to the presence of inducible ischemia, implicating either scar or artifact as the cause.

**Figure 7:**
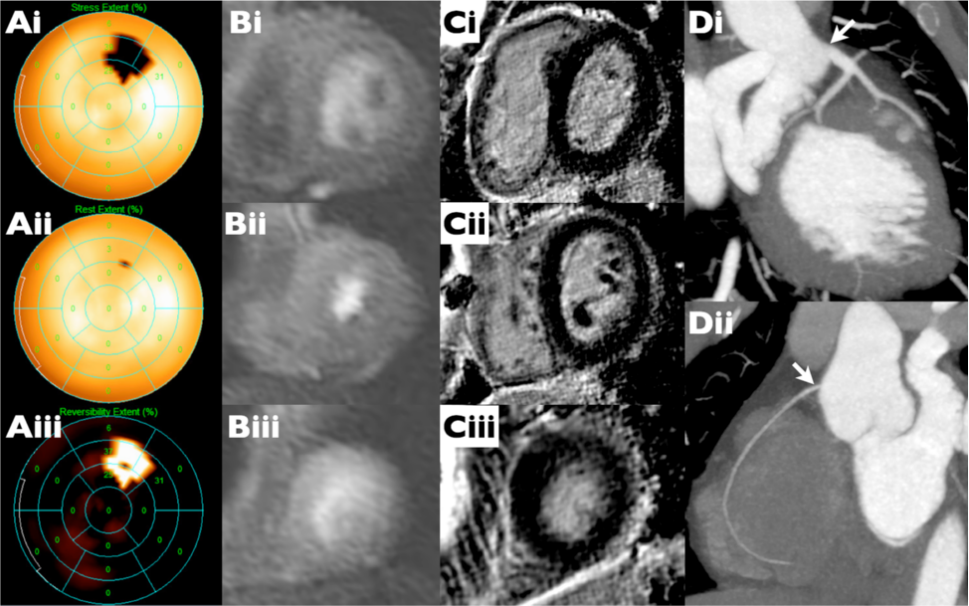
**Example of a false positive SPECT with moderate ‘inducible ischemia’. (Ai-ii)** MIBI bulls eye plots demonstrating a defect at stress in the basal to mid anterolateral wall **(i)** which appears fully reversible at rest **(ii)** with a SDS of 5 **(iii)** suggesting a moderate inducible perfusion defect. **(Bi-iii)** Basal, mid and apical short axis slices from stress CMR show visually normal perfusion in all territories. Quantitative perfusion (not shown) confirmed normal stress perfusion values at the sites of the defect seen on MIBI. **(Ci-iii)** Basal, mid and apical short axis slices from LGE stack demonstrate lack of any myocardial scar. **(Di-ii)** CT coronary angiogram performed in view of the discordance between SPECT and CMR shows that the coronary arteries are entirely normal.

In the remaining 8 cases of disagreement the SDS scores were 2 in four patients, SDS 3 in two patients, SDS 4 in one patient and SDS 5 in the final case (Figure 7). In 6 out of these 8 cases CMR demonstrated normal coronary ostia, perfusion and absent LGE suggesting that the SPECT defects were artifactual. In the two other cases, a small amount of LGE was present in one case (patient 7); in the other case (patient 22), proximal RCA kinking was called, with a small SPECT perfusion defect in the LAD territory that was felt to be artifactual in view of the normal ostial left main appearances and absence of LGE in the anterolateral wall (Figure 4).

## Discussion

In this observational study of stable adult patients with prior ASO, none had evidence of stress -induced perfusion deficit by CMR, neither visually nor with fully quantitative measurement of blood flow. We further demonstrated a low incidence of overt focal myocardial fibrosis, which principally related to enhancement in the region of prior ventricular septal defect repair rather the intrinsic myocardial injury. Similarly, we were able to visualize the coronary ostia with a high degree of success (24/27 cases) and in no case did coronary CMR show potential proximal luminal obstruction when images were analysed with centre-line reformatting. Importantly, in the one case where there was significant clinical concern regarding ischemic chest pain there was no evidence of perfusion abnormality, myocardial scar or coronary distortion by CMR - corresponding to subsequent normal invasive coronary angiography.

We have demonstrated the feasibility of using CMR with dynamic stress perfusion, ventricular function, coronary artery and scar imaging to provide comprehensive assessment. Since the standard of care at our institution (and at the Labatt Family Heart Centre at SickKids, Toronto) has historically involved the use of radio-isotopes (SPECT), our study also includes data from this method. Our cohort underwent both stress SPECT and CMR examinations under identical physiologic conditions since a single episode of stress with the long-acting vasodilator dipyridamole allowed sequential delivery of both Sestamibi™ and gadolinium tracers while the patient remained on the magnet table. The relatively long half-life of technetium made it possible to then acquire the stress SPECT images back in the nuclear laboratory once the remainder of the CMR study was complete.

### Coronary complications in adults after the arterial switch procedure

In the current era, coronary complications including coronary-related deaths late after ASO are rare events in both children and adults [4]. In historical cohorts, however, these complications have been described by several series and case reports [12-15] associated with coronary distribution as an independent risk factor [15]. The major problem with those historical surgical series was the common use of the single ostium technique for coronary transfer, which was shown to have a high rate of coronary occlusion and therefore has now been abandoned. Although a single right coronary artery was an independent risk factor for cardiovascular events in one study [15], in the current era coronary anatomy no longer determines outcome [4]. Recent work seems to confirm a genuinely low rate of significant coronary complications - at least in early adult life [1,15,16]. In these series, none of the adults suffered acute coronary syndrome during follow-up.

The vast majority of our cohort had had the ASO in the current era (after 1986). In line with the low prevalence of late coronary complication, our data confirm the absence of significant proximal coronary abnormality following reimplantation. We also demonstrate a reassuringly low prevalence of scar despite prior cardiopulmonary bypass. Perfusion was uniformly normal on a qualitative basis but we did detect a mild increase in resting flow in some patients – a finding of unknown significance, which replicates that of earlier PET data [17]. As such, measured coronary flow reserve for the group was towards the lower end of the accepted normal range for CMR perfusion.

### Assessment for silent myocardial ischemia after the arterial switch procedure

Historically, nuclear perfusion techniques were commonly employed in the follow-up of children and adults following ASO surgery in our center. However they may not be ideal in this population. Firstly, they expose radiosensitive young adults to a significant dose of ionizing radiation. Secondly, many post-operative patients have left bundle branch block, creating perfusion defects in the absence of obstructive disease or myocardial scar. Thirdly, patients who underwent cardiopulmonary bypass as neonates may have sustained myocardial damage as a result of inadequate myocardial protection creating perfusion defects that are not linked to the longer-term patency of the translocated coronary arteries [18,19]. Resting perfusion abnormalities are very common in this population and show little correlation to symptoms or exercise-induced ECG abnormalities [20].

Stress perfusion CMR has been extensively validated in the adult ischemic heart disease population [21]. It has been shown to be superior to SPECT [7] and to have comparable diagnostic accuracy to both PET [22] and fractional flow reserve [23]. By comparison, the literature on use of stress CMR in congenital heart disease populations is sparse [24,25]. The study by Manso et al. [26] employed stress perfusion CMR to look at survivors of the ASO procedure in a younger age group (median age of 14 years). In keeping with our results, they found no evidence of visible perfusion abnormality but did not seek to quantitatively assess absolute myocardial blood flow. Manso et al. also failed to find late gadolinium enhancement in the myocardium - providing further indirect support for our contention that perfusion defects seen on SPECT but unmatched by scar on late gadolinium enhancement imaging are most likely related to artifact.

Although ostial obstrucion has been described as the main reason for late onset myocardial infarction, other mechanism leading to myocardial ischemia may be discussed in the setting of prior ASO. After the Lecompte maneuvre, compression of the coronary arteries by the pulmonary arteries may occur and in this case, exercise could induce ischemia [14]. In this scenario, pharmacological stress testing may not trigger the underlying mechanism for ischemia and perfusion CMR may have insufficient sensitivity.

### Clinical outlook

Although our data are reassuring, the ASO has only been in widespread use since the 1980s and so the ultimate fate of the coronary arteries after ASO remains uncertain. At the current time there is no single accepted surveillance protocol for this patient group [22,27,28]. Notwithstanding the facts that patients with coronary denervation following ASO may not experience typical symptoms of angina, and the finding of a high prevalence of coronary atheroscleriosis by IVUS examination in a historical cohort of children with prior ASO [29], our data do not support routine and serial imaging screening for silent myocardial ischemia in asymptomatic adults after ASO. Nevertheless, we emphasize that a comprehensive evaluation by CMR - without perfusion protocol - is recommended as a baseline assessment in these patients when they present in adulthood in order to assess: coronary anatomy and the pulmonary artery tree; to rule out ostial obstruction; to evaluate for unexpected prior myocardial injury; and to determine biventricular function and volumes. In specific cases where coronary obstruction is present, or compression of the coronary artery by the pulmonary artery is suggested, then we recommend further functional ischemic testing, either with perfusion CMR in the first or with exercise SPECT in the latter scenario.

### Study limitations

This was a single center study from a relatively narrow surgical era later on in the operative experience for the arterial switch procedure with a limited number of patients. Nonetheless our results are congruent with the lack of published adverse outcomes in adult ASO patients and suggest that coronary anatomical concerns may have been over-stated in an asymptomatic adult population. Almost all of the patients in our cohort underwent imaging for surveillance rather than symptomatic chest pain or breathlessness and therefore we lack confirmation of coronary normality by coronary artery catheterization in most patients. However stress perfusion CMR has been shown to have a very high degree of diagnostic accuracy compared to PET, IVUS or quantitative coronary angiography and has previously been shown to be superior to SPECT in the context of ischemic heart disease [7]. Although myocardial flow reserve obtained by quantitative perfusion CMR was comparable to prior published data, these values should be interpreted with caution as data from a healthy control group is lacking. None of our patients have had any adverse coronary events since recruitment into the study over a median follow up period of 24 months. However we accept that we cannot exclude a potential for accelerated atherosclerosis at a later time point in the natural history of operated *d-*transposition of the great arteries.

## Conclusions

Adult ASO survivors in this study had no CMR evidence of myocardial ischemia, scar or coronary ostial abnormality. Compared to SPECT, CMR provides additional valuable information about the coronary artery anatomy. The data show that the asymptomatic and clinically stable adult ASO patient has a low pre-test probability for inducible ischemia. In this situation, it is likely that routine evaluation with stress CMR is unnecessary.

## Abbreviations

ASO: Arterial switch operation
CMR: Cardiovascular magnetic resonance
CT: Computed tomography
LGE: Late gadolinium enhancement
PET: Positron emission tomography
SDS: Summed difference scores
SPECT: Single-photon emission computed tomography
SRS: Summed rest scores
SSS: Summed stress scores
